# Overexpression of miR-100-5p inhibits papillary thyroid cancer progression via targeting FZD8

**DOI:** 10.1515/med-2022-0490

**Published:** 2022-07-06

**Authors:** Peng Ma, Jianli Han

**Affiliations:** Department of Thyroid Surgery, Shanxi Bethune Hospital, Shanxi Academy of Medical Sciences, Tongji Shanxi Hospital, Third Hospital of Shanxi Medical University, Taiyuan 030032, Shanxi Province, P.R. China; Department of Thyroid Surgery, Shanxi Bethune Hospital, Shanxi Academy of Medical Sciences, Tongji Shanxi Hospital, Third Hospital of Shanxi Medical University, No. 99 Longcheng Street, Taiyuan 030032, Shanxi Province, P.R. China

**Keywords:** papillary thyroid cancer, cell proliferation, cell apoptosis, miR-100-5p, Frizzled Class Receptor 8

## Abstract

Papillary thyroid cancer (PTC) is the most prevalent type of TC worldwide; however, its pathological process remains unclear at the molecular level. In the current study, we analyzed the microarray data of PTC tissues and non-neoplastic thyroid tissues, and confirmed miR-100-5p as a downregulated miRNA in PTC. Via bioinformatic approach, western blotting, and TOP/FOP-flash assay, miR-100-5p was observed to be involved in the inactivation of Wnt/β-catenin signaling in TPC-1 and KTC-1. Frizzled Class Receptor 8 (FZD8), the coupled receptor for canonical Wnt/β-catenin signaling, was verified to be targeted and inhibited by miR-100-5p in TPC-1 and KTC-1. In the function assay, miR-100-5p mimic repressed PTC cell proliferation and induced cell apoptosis of TPC-1 and KTC-1; meanwhile, transfection of full-length FZD8 attenuated the effect of miR-100-5p mimic. Moreover, in the collected samples, miR-100-5p was lowly expressed in PTC tissues compared with normal tissues, especially in those of advanced stage (Stage III/IV vs Stage I/II), while FZD8 was highly expressed in PTC tissues, which in PTC tissues was inversely correlated to miR-100-5p. Thus, we suggest that overexpression of miR-100-5p inhibits the development of PTC by targeting FZD8.

## 1 Introduction

Thyroid cancer (TC) was one of the top ten cancer types for estimated new cases globally for both sex in 2020 [1]. Statistics indicate that TC is relatively more prevalent in Eastern Asia, North America, and Micronesia/Polynesia compared to other regions in the world [1]. Most TC (85%) are originated from thyroid follicular cells, and TC can be further divided into papillary thyroid cancer (PTC, 75%), follicular thyroid cancer (FTC, 15%), and Hürthle cell cancer (HCC, 10%) [2]. Generally, the 5-year survival rate of patients with PTC is high when diagnosed early [3]; however, the recurrence happens to a small portion of them and some of them are under the threat of death upon cancer cell metastasis [4]. Surgery is the most commonly used treatment for patients with PTC. Owing to decades of basic research, several novel targets have been discovered for PTC and data from clinical research suggest their effectiveness in the treatment of PTC [5,6]. It is urgent to further explore the molecular mechanism of PTC.

MicroRNAs (miRNAs) are defined as small non-coding RNAs (21–25 nt) [7]. One miRNA regulates its target mRNA genes via directly binding to the 3′ untranslated region (3′UTR) [8]. Due to critical roles of targets in regulating signaling network in cells, the dysregulation of miRNAs is highly related with numerous human diseases, e.g., cancer [9,10]. The oncogenic and anti-cancer effect of several miRNAs have been reported in PTC, including miR-3126-5p [11], miR-1179, miR-133b, miR-3194, miR-3912, miR-548j, miR-6720, miR-6734, miR-6843 [12], and miR-363-3p [13]. However, the functions of many dysregulated miRNAs have not been studied yet.

In the current study, miR-100-5p was screened out as a downregulated miRNA in PTC. Our assays further showed that, miR-100-5p inhibited cell proliferation and induced cell apoptosis of PTC cells by targeting Frizzled Class Receptor 8 (FZD8) and followed by inactivation of Wnt/β-catenin signaling in PTC.

## 2 Materials and methods

### 2.1 Human clinical tissues

In total, 50 human PTC samples and the adjacent normal samples were collected from Shanxi Bethune Hospital during 2017 and 2020. None of them received anti-cancer therapy prior to the surgery. The samples were stored in liquid nitrogen for further extraction of RNA. Current study was approved by the Research Ethics Committee of Shanxi Bethune Hospital. The written informed consents were provided by all participants.

### 2.2 Cell culture, RNA oligonucleotides, and plasmids

Two PTC cell lines (TPC-1, KTC-1) and the immortalized thyroid follicular epithelium cell line (Nthy-ori3-1) were obtained from Chinese Academy of Sciences, Shanghai Institute of Biochemistry and Cell Biology (Shanghai, China). Cells were kept in RPMI1640 supplemented with 10% FBS and 1% penicillin/streptomycin at 37°C in an incubator.

miR-NC mimic and miR-100-5p mimic were obtained from Genepharma (Shanghai, China). Cells were transfected with the RNA oligonucleotides via Lipofectamine RNAiMax (Invitrogen; Thermo Fisher, Carlsbad, CA, USA).

pcDNA3.1 was obtained from Invitrogen. pcDNA3.1-FZD8 was constructed by inserting full length FZD8 into pcDNA3.1 vector. TOPFlash and FOPFlash plasmids were bought from YouBio (Changsha, China). pmirGLO was the product of Promega Corp. (Madison, WI, USA). FZD8 3′UTR or its mutant form was ligated into pmirGLO to obtain pmirGLO-FZD8 3′UTR and pmirGLO-FZD8 3′UTR Mutant, respectively, which was transfected into cells by Lipofectamine 2000 (Invitrogen).

### 2.3 CCK8 and flow cytometry assay

Cell proliferation was measured by a Cell Counting Kit (CCK-8, Dojindo, Tokyo, Japan). TPC-1 and KTC-1 (5 × 10^3^) were seeded in wells in 96-well plates. On day 1, 2, and 3 following plating, 10 μL of CCK-8 solution was mixed with the fresh medium and replaced the medium in the wells. After 4 h, the medium was scanned by a Microplate Reader and OD 450.

Flow cytometry was utilized to measure the percent of apoptotic TPC-1 and KTC-1 cells. Cells were stained by Annexin V-FITC and PI (Annexin V-FITC cell apoptosis detection kit, Beyotime, Shanghai, China), and analyzed by the flow cytometry (FACSCanto II, BD Biosciences, Franklin Lakes, NJ, USA). The data were analyzed by FlowJo. The cells were divided into live, early apoptosis, late apoptosis, and necrosis groups.

### 2.4 Western blotting

Total proteins from cells were extracted by RIPA (Thermo Fisher). BCA kit (Thermo Fisher) was utilized to measure the concentration of lysates. In brief, 20 μg protein was electrophoresed on the SDS-PAGE followed by transferring to the polyvinylidene fluoride membrane, which was treated by the primary and secondary antibody sequentially. Images of bands were acquired via development of ECL Western Blotting Substrate (Thermo Fisher) and a ChemiDoc XRS system (Bio-Rad, Carlsbad, CA, USA). The antibody information was listed as follows: FZD8 (ab150500, Abcam, Cambridge, UK), β-actin (AA128, Beyotime), Myc (H00004609-D01, Abnova, Taipei, China), Cyclin D1 (H00000595-D01, Abnova), anti-mouse antibody (PAB9346, Abnova), and anti-rabbit antibody (MAB19500, Abnova).

### 2.5 RT-PCR

Total RNA from cells and tissue samples was extracted by TRIzol (Invitrogen). Nanodrop 2000 was used to determine the RNA concentration and quality. PrimeScript^®^ miRNA cDNA Synthesis kit (TaKaRa, Tokyo, Japan) and PrimeScript^®^ RT reagent kit (TaKaRa) were utilized to conduct reverse transcription of miRNA and general genes, respectively. TB Green^®^ Fast qPCR Mix was used to perform RT-PCR. The condition was: Step 1, 95°C (30 s); Step 2, 95°C (5 s), 60°C (10 s), 35 cycles. U6 and β-actin served as references for miRNA and mRNA, respectively. 2^−ΔΔCt^ was used for data analysis.

### 2.6 Dual luciferase reporter assay

The cells were transfected with pmirGLO in combination with RNA oligonucleotide. At 48 h following transfection, cells were collected and subjected to lysate with reagent in Dual Luciferase Reporter Assay System kit (Promega Corp.). Luciferase activity was measured on a GloMax luciferase detector (Promega Corp.).

### 2.7 TOPFlash/FOPFlash assay

Cells were transfected with TOPFlash and FOPFlash vector in combination with RNA oligonucleotide. On day 2 following transfection, they were treated with reagent from Dual Luciferase Reporter Assay System kit (Promega Corp.) and measured on the GloMax luciferase detector (Promega Corp.).

### 2.8 Bioinformatic analysis

Microarray data of GSE104006 (five non-neoplastic thyroid and 29 PTC tissues) were obtained from GEO database (https://www.ncbi.nlm.nih.gov/geo/). Those differentially expressed miRNAs were analyzed by GEO2R (https://www.ncbi.nlm.nih.gov/geo/geo2r/) from GEO database. The targets for miR-100-5p were predicted using PITA (https://genie.weizmann.ac.il/pubs/mir07/mir07_prediction.html), TargetScan 7.2 (http://www.targetscan.org/vert_72/), and miRanda (http://www.microrna.org/microrna/home.do). The conserved binding sites were analyzed using TargetScan 7.2 (http://www.targetscan.org/vert_72/).

### 2.9 Statistical analyses

The data were analyzed by GraphPad Prism 6.0 and expressed as mean  ±  SD. Student’s *t*-test and one-way ANOVA were performed in two and three groups, respectively. Student–Newman–Keuls (S–N–K) method was selected as post-analysis for one-way ANOVA. *p* < 0.05 was statistical significance. Each experiment was repeated three independent times.

## 3 Results

### 3.1 miR-100-5p was a decreased miRNA in PTC

**Figure 1:**
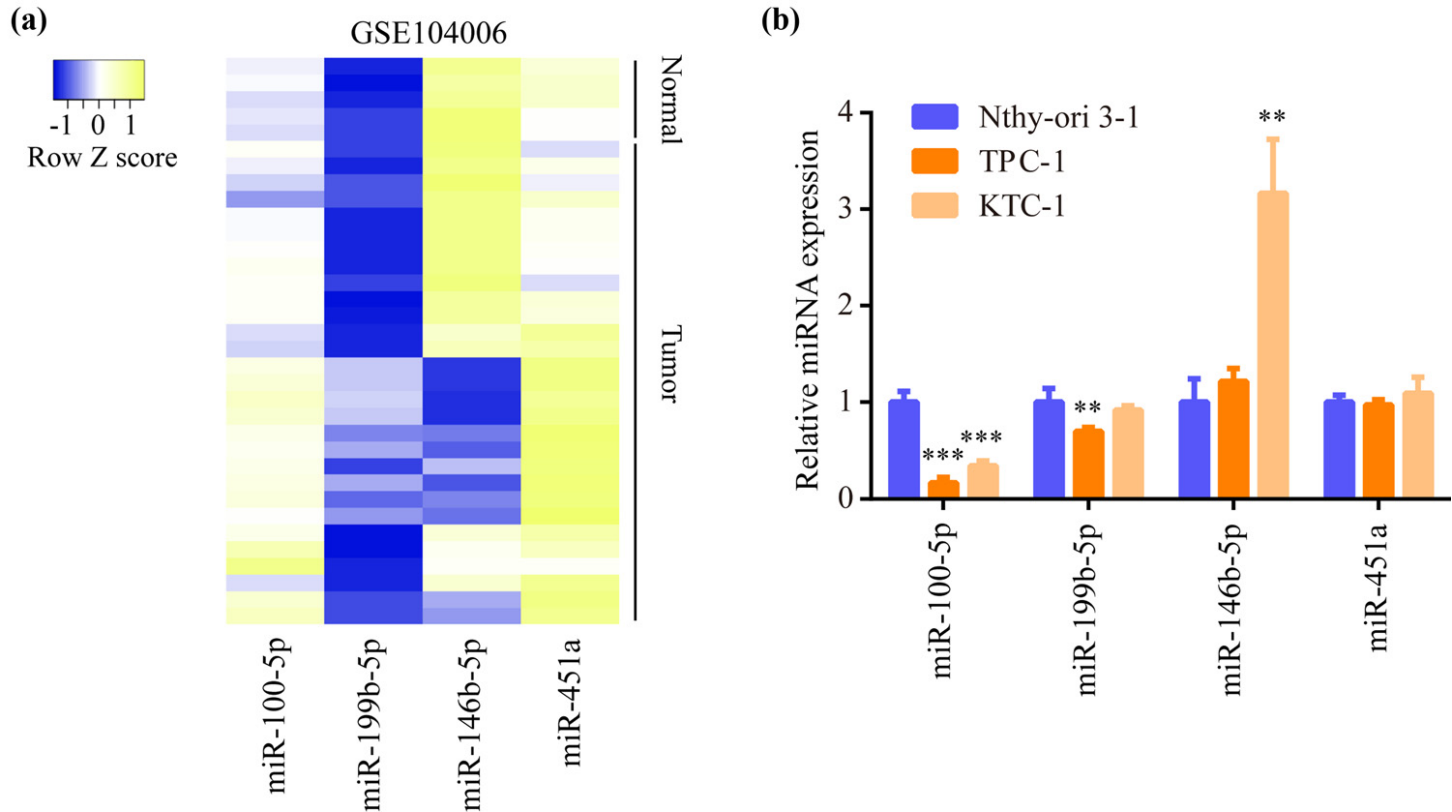
miR-100-5p was one of the differentially expressed miRNAs in PTC: (a) Microarray data of five non-neoplastic thyroid tissues and 29 PTC tissues (GSE104006) were downloaded, and the differentially expressed miRNAs were analyzed. Heatmap showed the top four differentially expressed miRNAs between two groups. (b) RT-PCR exhibited the expression difference of miR-100-5p, miR-199b-5p, miR-146b-5p, and miR-451a among TPC-1, KTC-1, and Nthy-ori3-1. **, *p* < 0.01 and ***, *p* < 0.001.

To explore PTC related miRNAs, we retrieved the expression data from a microarray of five non-neoplastic thyroid tissues and 29 PTC tissues. The top four differentially expressed miRNAs were miR-100-5p (downregulation), miR-199b-5p (downregulation), miR-146b-5p (upregulation), and miR-451a (downregulation) (Figure 1a). For validation, we detected the expression of these miRNAs in TPC-1, KTC-1, and Nthy-ori3-1. Compared to Nthy-ori3-1, miR-199b-5p expression was specifically downregulated in TPC-1 but not in KTC-1, miR-146b-5p expression was specifically upregulated in KTC-1 but not in TPC-1, miR-451a expression was not significantly changed in KTC-1 or TPC-1, only miR-100-5p was strongly downregulated in both TPC-1 and KTC-1 (Figure 1b). Consequently, miR-100-5p was chosen as the research subject in the current study.

### 3.2 miR-100-5p inactivated Wnt/β-catenin signaling in PTC cells

**Figure 2:**
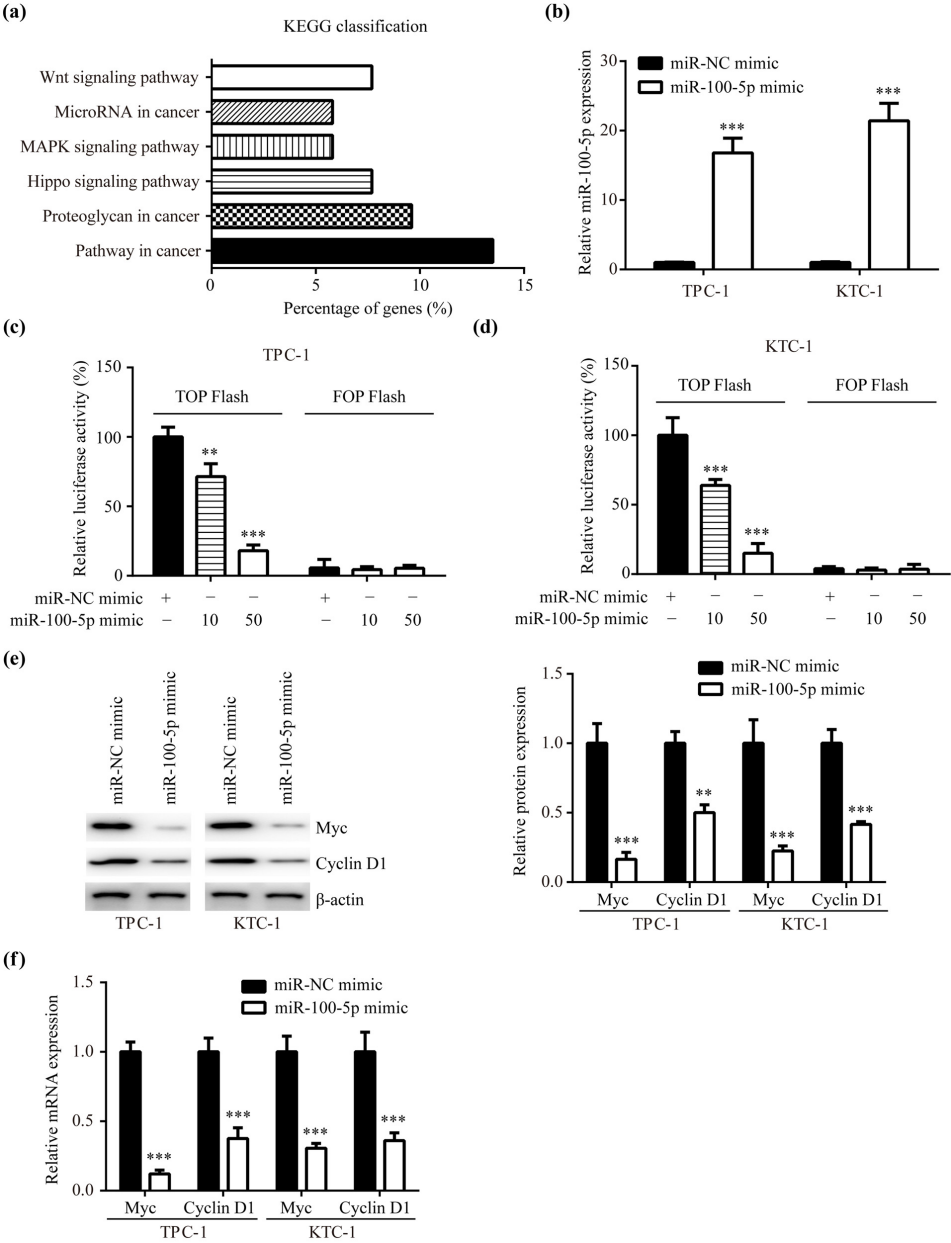
Wnt/β-catenin signaling was repressed by miR-100-5p in PTC: (a) KEGG pathway analysis was utilized for analyzing targets of miR-100-5p predicted by TargetScan7.2, (b) RT-PCR presented that miR-100-5p mimic elevated miR-100-5p in TPC-1 and KTC-1, (c and d) the TOPFlash/FOPFlash assay presented that miR-100-5p mimic reduced the activity of Wnt/β-catenin pathway in TPC-1 and KTC-1 in a dose-dependent manner, (e) western blotting revealed that miR-100-5p decreased Myc and Cyclin D1 protein levels in TPC-1 and KTC-1, and (f) RT-PCR revealed that miR-100-5p decreased Myc and Cyclin D1 mRNA levels in TPC-1 and KTC-1. **, *p* < 0.01 and ***, *p* < 0.001.

To study the downstream molecules of miR-100-5p, we used TargetScan7.2 to search them (Table A1). By KEGG pathway analysis, these targets were mainly involved in several signaling pathways including Wnt/β-catenin pathway, MAPK pathway, and Hippo pathway (Figure 2a). In comparison with MAPK pathway and Hippo pathway, there were relatively much more genes enriched in Wnt/β-catenin pathway, which was selected as the research subject in the current study. More importantly, Wnt/β-catenin signaling remains one of the most well-characterized oncogenic pathways in PTC [14–16].

Then, we performed TOPFlash/FOPFlash assay to examine the activity of Wnt/β-catenin signaling. After verification of miR-100-5p overexpression in TPC-1 and KTC-1 by transfection of miR-100-5p mimic (Figure 2b), miR-100-5p mimic was observed to decrease TOPFlash luciferase in TPC-1 and KTC-1 in a dose-dependent manner (Figure 2c and d). Moreover, mRNA and protein levels of Myc and Cyclin D1, two Wnt/β-catenin target genes [17], were also significantly decreased by miR-100-5p mimic in TPC-1 and KTC-1 (Figure 2e and f).

### 3.3 miR-100-5p targeted FZD8 in PTC cells

**Figure 3:**
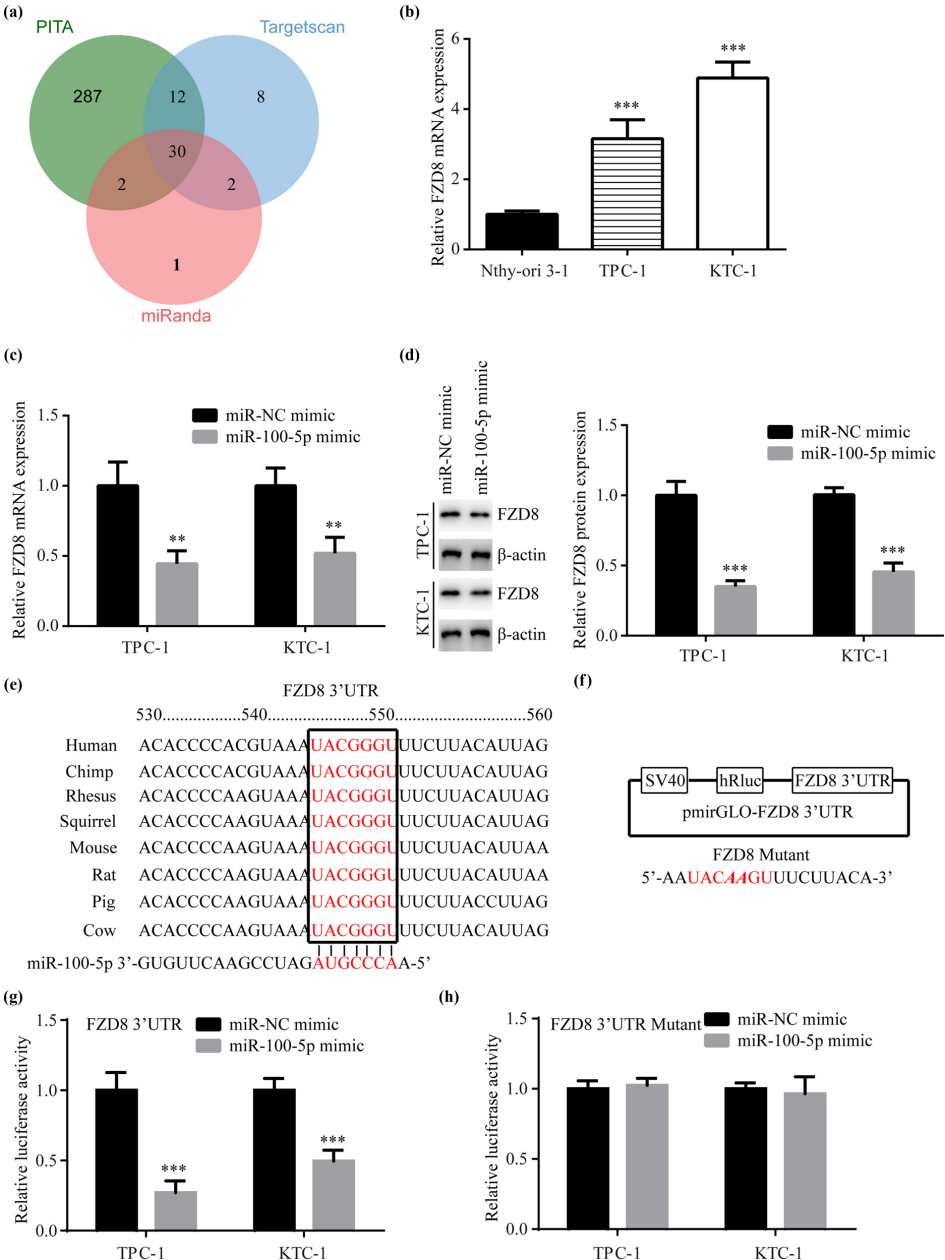
FZD8 was a target for miR-100-5p: (a) the targets of miR-100-5p were predicted using PITA, TargetScan7.2, and miRanda. The overlapping of the targets is presented in a vein map, (b) RT-PCR showed that FZD8 mRNA levels were increased in TPC-1 and KTC-1 compared to Nthy-ori3-1, (c) RT-PCR presented that miR-100-5p mimic decreased FZD8 mRNA levels in TPC-1 and KTC-1, (d) western blotting revealed that miR-100-5p mimic decreased FZD8 protein levels in TPC-1 and KTC-1, (e) the conserved binding sites for miR-100-5p on FZD8 mRNA are presented, (f) FZD8 3′UTR and its mutant form was inserted into pmirGLO plasmid, respectively, and (g and h) luciferase assay revealed that miR-100-5p reduced luciferase activity of FZD8 3′UTR but not FZD8 3′UTR Mutant in TPC-1 and KTC-1. **, *p* < 0.01 and ***, *p* < 0.001.

To explore the potential targets of miR-100-5p, we used three bioinformatic tools, including PITA, TargetScan7.2, and miRanda. In total, 30 targets were overlapped among three tools (Figure 3a).

Among the 30 targets, FZD8 which is the receptor for Wnt proteins [18] attracted our attention. Upon binding between Wnt and FZD8, FZD8 can activate β-catenin dependent/independent signals [17]. However, the relationship between miR-100-5p and FZD8 in PTC has not been studied yet.

Later, we observed that FZD8 mRNA level was increased in TPC-1 and KTC-1 compared to Nthy-ori3-1 (Figure 3b). In TPC-1 and KTC-1, miR-100-5p mimic significantly decreased FZD8 mRNA and protein levels (Figure 3c and d). The binding site of FZD8 for miR-100-5p was conserved among species (Figure 3e). We next inserted FZD8 3′UTR in pmirGLO luciferase reporter vector (Figure 3f). In TPC-1 and KTC-1, the luciferase activity of FZD8 3′UTR was decreased by miR-100-5p mimic (Figure 3g), but FZD8 3′UTR Mutant was not significantly changed (Figure 3h).

### 3.4 miR-100-5p repressed PTC cell proliferation and promoted PTC cell apoptosis via targeting FZD8

**Figure 4:**
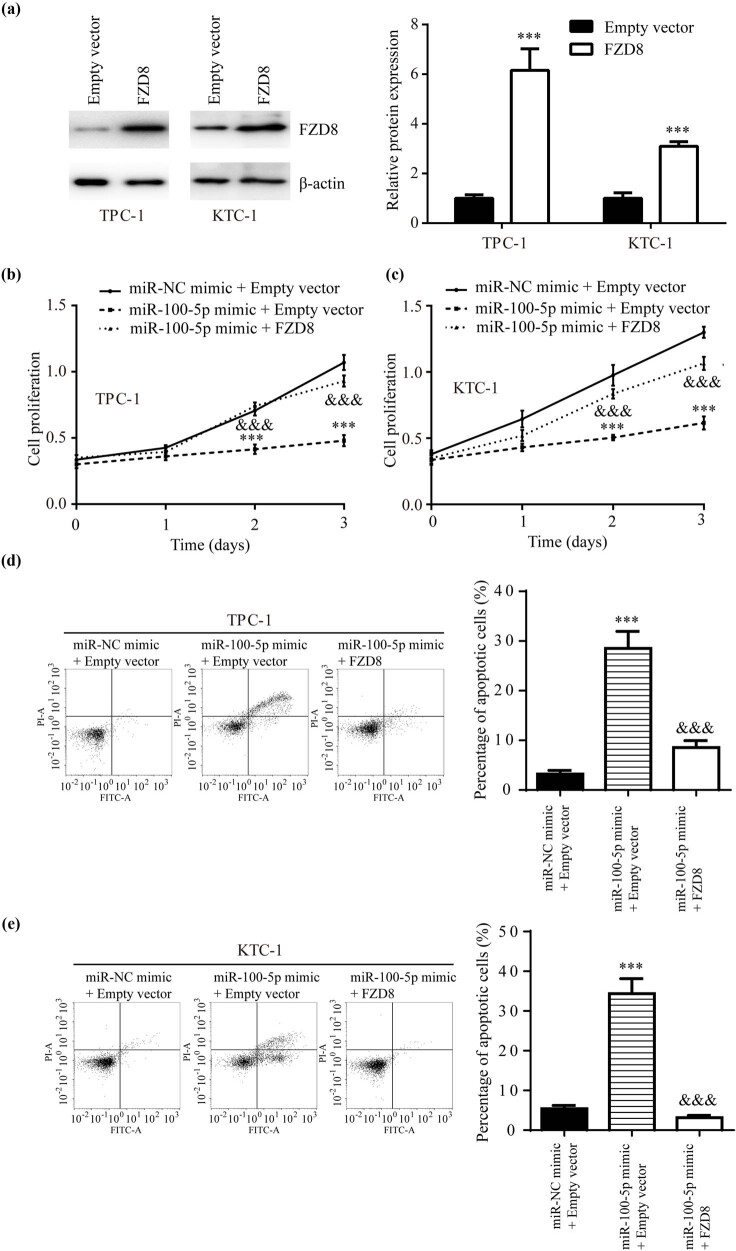
miR-100-5p/FZD8 regulated PTC cell proliferation and apoptosis: (a) western blotting showed that transfection of full length FZD8 increased FZD8 protein expression in TPC-1 and KTC-1, (b and c) the CCK8 assay revealed that miR-100-5p mimic inhibited cell proliferation, this effect was attenuated by FZD8 in TPC-1 and KTC-1, and (d and e) flow cytometry analysis presented that miR-100-5p mimic promoted cell apoptosis and this effect was attenuated by FZD8 in TPC-1 and KTC-1. ***, vs miR-NC mimic + Empty vector, *p* < 0.001; &&&, vs miR-100-5p mimic + Empty vector, *p* < 0.001.

Transfection of full length FZD8 increased FZD8 protein expression in TPC-1 and KTC-1 (Figure 4a). Overexpression of miR-100-5p significantly impaired TPC-1 cell proliferation, this effect was rescued by FZD8 overexpression (Figure 4b). Similar results were observed in KTC-1 (Figure 4c). Via flow cytometry, we found that miR-100-5p mimic significantly evoked cell apoptosis and this effect was reversed via FZD8 overexpression in TPC-1 and KTC-1 (Figure 4d and e).

### 3.5 miR-100-5p was negatively correlated with FZD8 in PTC tissues

**Figure 5:**
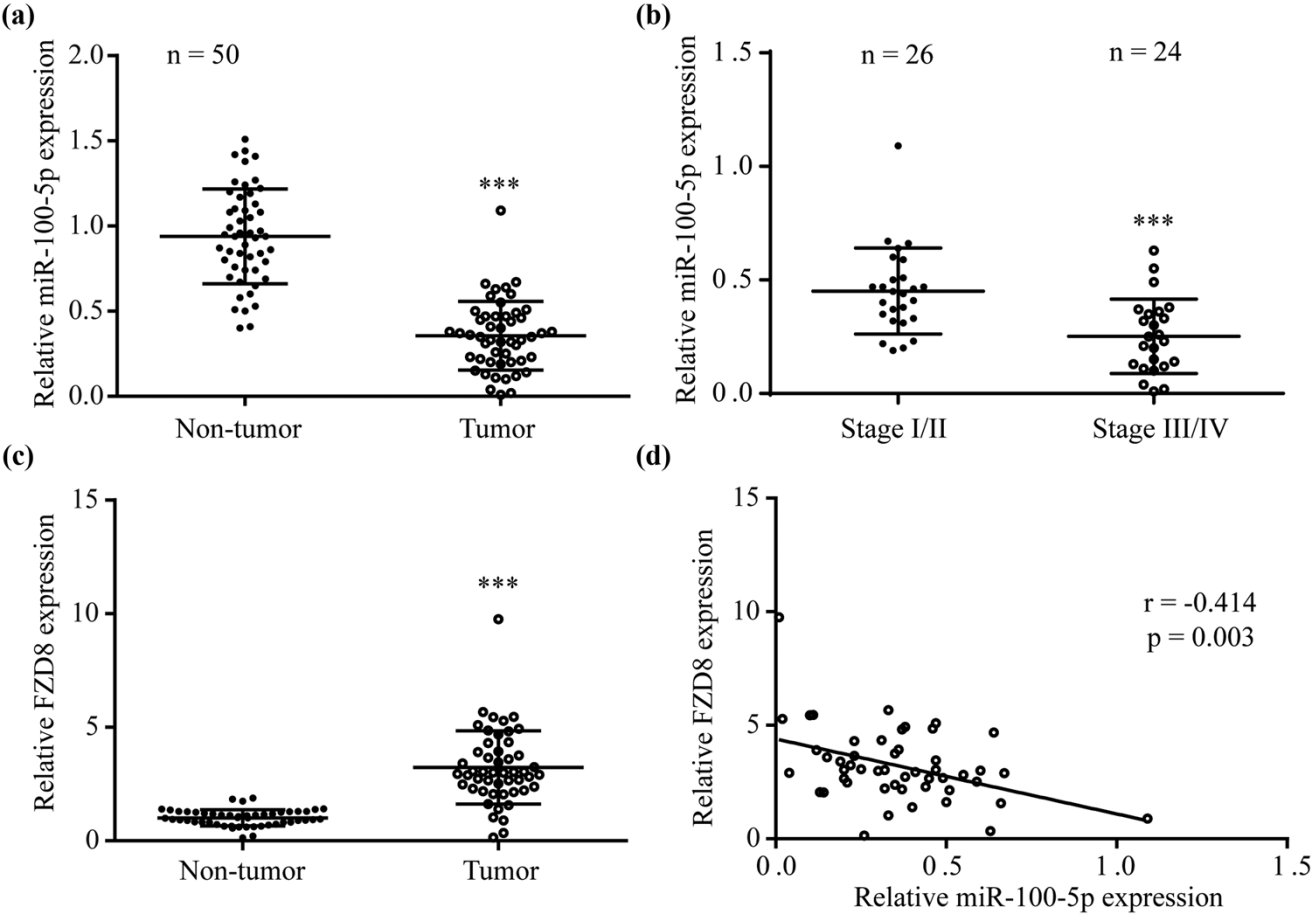
miR-100-5p was inversely associated to FZD8 in PTC: (a) RT-PCR showed that miR-100-5p was lower in PTC tissues than non-tumors tissues from 50 patients with PTC, (b) miR-100-5p levels were relatively lower in tumors at Stage III/IV than those at Stage I/II, (c) RT-PCR showed that FZD8 mRNA expression was higher in PTC tissues than non-tumors tissues from 50 patients with PTC, and (d) the Pearson correlation analysis indicated an inverse correlation between miR-100-5p and FZD8 in PTC tissues. ***, *p* < 0.001.

In the collected clinical samples in the current study, RT-PCR displayed a significant decrease of miR-100-5p in PTC tissues compared to the matched non-tumor tissues (Figure 5a). In addition, lower miR-100-5p levels were found from tumors of advanced stage compared with those of early stage (Figure 5b). On the contrary, FZD8 mRNA levels were elevated in PTC tissues compared to the matched non-tumor tissues (Figure 5c). The Pearson correlation analysis indicated that miR-100-5p was inversely (*r* = −0.414) correlated to FZD8 in these PTC tissues (Figure 5d).

## 4 Discussion

Two decades of studies have revealed multiple oncogenic pathways in PTC; however, the potential roles of miRNAs in regulating these pathways and their contribution to PTC remains poorly defined. The pro-cancer and anti-cancer functions of miR-100-5p have been shown from cancers of numerous origins, for example, miR-100-5p is lowly expressed in prostate cancer, which impairs cancer cell proliferation, migration, and invasion via downregulating mTOR [19]; in contrast, overexpression of miR-100-5p is observed in ovary cancer, which promotes cell invasion via directly targeting SMARCD1 [20]. In the present study, by analyzing previously published microarray data, miR-100-5p was identified as one of the most significantly downregulated miRNAs in PTC. The experiments confirmed that miR-100-5p was decreased in PTC cells and PTC tissues. miR-100-5p mimic repressed cell proliferation and induced cell apoptosis in PTC cells. Thus, the data implied that miR-100-5p functioned as a tumor suppressor in PTC for the first time. However, the potential molecules responsible for the function of miR-100-5p remain undiscovered.

By KEGG pathway analysis and TOPFlash/FOPFlash assay, miR-100-5p was discovered to inactivate Wnt/β-catenin signaling in PTC cells. Regarding Wnt/β-catenin signaling, its overactivation results in PTC cell proliferation, resistance to cell apoptosis, cell invasion ,and drug resistance [14–16]. However, the potential molecules which link miR-100-5p and Wnt/β-catenin signaling remain undiscovered.

Studies have revealed that many key positive and negative modulators for Wnt/β-catenin signaling are targets of miRNAs, and dysregulation of miRNAs is one of the most crucial mechanisms for Wnt/β-catenin signaling activation, e.g., Bai et al. report that miR-150 targets RAB11A to inactivate Wnt/β-catenin signaling in PTC [21]. Li et al. demonstrate that upregulation of miR-320a elevates ANRIL to inactivate Wnt/β-catenin signaling in PTC [22]. Accordingly, current study aimed to explore the potential mRNA targets for miR-100-5p.

Among the 30 targets of miR-100-5p predicted by PITA, TargetScan7.2, and miRanda, FZD8, which can activate β-catenin dependent/independent signals by serving as receptor for Wnt proteins [17,18], is selected as the research subject. Afterwards, overexpression of FZD8 was observed in TPC-1 and KTC-1 compared to Nthy-ori3-1, indicating the oncogenic role of FZD8 in PTC, which was in consistent with the function of FZD8 in other cancer types, including prostate cancer [23], non-small cell lung cancer [24], and head and neck squamous carcinomas [25]. Moreover, Chen et al. reported that FZD8 was regulated by circRNA_NEK6/miR-370-3p, and contributed to the progression of TC [26]. Very recently, Mao et al. reported that FZD8 was regulated by circRPS28 (hsa_circ_0049055)/miR-345-5p, contributed to promotion of cell growth and blockage of cell apoptosis of PTC [27].

Thereafter, FZD8 was shown to harbor conserved binding sites for miR-100-5p, and miR-100-5p acted as a novel regulator of FZD8. Additionally, in PTC cells, the biological function of miR-100-5p mimic was attenuated by overexpression of FZD8. Moreover, in the PTC samples, the negative correlation between miR-100-5p and FZD8 was discovered. The data added novel insights for understanding the complex signaling transduction in PTC.

Collectively, data from clinical samples and cell-based assays indicated that miR-100-5p was functionally significant for PTC. Targeting miR-100-5p may be a promising treatment for patients with PTC.

However, there was a limitation in the current study, among the three signaling pathways, the present study only studied the relation between miR-100-5p and Wnt/β-catenin signaling, but not MAPK or Hippo, which will be investigated in our future work.

## Supplementary Material

Supplementary Table

## References

[1] Sung H, Ferlay J, Siegel RL, Laversanne M, Soerjomataram I, Jemal A, et al. Global cancer statistics 2020: GLOBOCAN estimates of incidence and mortality worldwide for 36 cancers in 185 countries. CA Cancer J Clin. 2021;71(3):209–49. 10.3322/caac.21660 33538338

[2] Cabanillas ME, Ryder M, Jimenez C. Targeted therapy for advanced thyroid cancer: kinase inhibitors and beyond. Endocr Rev. 2019;40(6):1573–604. 10.1210/er.2019-00007 PMC7341904 31322645

[3] Paricharttanakul NM, Saharat K, Chokchaichamnankit D, Punyarit P, Srisomsap C, Svasti J. Unveiling a novel biomarker panel for diagnosis and classification of well-differentiated thyroid carcinomas. Oncol Rep. 2016;35(4):2286–96. 10.3892/or.2016.4567 26782318

[4] Abdullah MI, Junit SM, Ng KL, Jayapalan JJ, Karikalan B, Hashim OH. Papillary thyroid cancer: genetic alterations and molecular biomarker investigations. Int J Med Sci. 2019;16(3):450–60. 10.7150/ijms.29935 PMC6428975 30911279

[5] Capdevila J, Trigo JM, Aller J, Manzano JL, Adrián SG, Llopis CZ, et al. Axitinib treatment in advanced RAI-resistant differentiated thyroid cancer (DTC) and refractory medullary thyroid cancer (MTC). Eur J Endocrinol. 2017;177(4):309–17. 10.1530/EJE-17-0243 28687563

[6] Falchook GS, Millward M, Hong D, Naing A, Piha-Paul S, Waguespack SG, et al. BRAF inhibitor dabrafenib in patients with metastatic BRAF-mutant thyroid cancer. Thyroid. 2015;25(1):71–7. 10.1089/thy.2014.0123 PMC4291160 25285888

[7] Shan G, Zhou X, Gu J, Zhou D, Cheng W, Wu H, et al. Downregulated exosomal microRNA-148b-3p in cancer associated fibroblasts enhance chemosensitivity of bladder cancer cells by downregulating the Wnt/β-catenin pathway and upregulating PTEN. Cell Oncol (Dordr). 2021;44(1):45–59. 10.1007/s13402-020-00500-0 PMC7906940 33423167

[8] Kondrotienė A, Daukša A, Pamedytytė D, Kazokaitė M, Žvirblienė A, Daukšienė D, et al. Plasma-derived miRNA-222 as a candidate marker for papillary thyroid cancer. Int J Mol Sci. 2020;21(17):6445. 10.3390/ijms21176445 PMC7503340 32899424

[9] Song Y, Zeng S, Zheng G, Chen D, Li P, Yang M, et al. FOXO3a-driven miRNA signatures suppresses VEGF-A/NRP1 signaling and breast cancer metastasis. Oncogene. 2021;40(4):777–90. 10.1038/s41388-020-01562-y PMC7843418 33262463

[10] Rupaimoole R, Slack FJ. MicroRNA therapeutics: towards a new era for the management of cancer and other diseases. Nat Rev Drug Discov. 2017;16(3):203–22. 10.1038/nrd.2016.246 28209991

[11] Li M, Chai HF, Peng F, Meng YT, Zhang LZ, Zhang L, et al. Estrogen receptor β upregulated by lncRNA-H19 to promote cancer stem-like properties in papillary thyroid carcinoma. Cell Death Dis. 2018;9(11):1120. 10.1038/s41419-018-1077-9 PMC6214949 30389909

[12] Yi W, Liu J, Qu S, Fan H, Lv Z. An 8 miRNA-based risk score system for predicting the prognosis of patients with papillary thyroid cancer. Technol Cancer Res Treat. 2020;19:1533033820965594. 10.1177/1533033820965594 PMC7570775 33054579

[13] Dong S, Xue S, Sun Y, Han Z, Sun L, Xu J, et al. MicroRNA-363-3p downregulation in papillary thyroid cancer inhibits tumor progression by targeting NOB1. J Investig Med. 2021;69(1):66–74. 10.1136/jim-2020-001562 PMC7803892 33077486

[14] Sastre-Perona A, Santisteban P. Role of the Wnt pathway in thyroid cancer. Front Endocrinol (Lausanne). 2012;3:31. 10.3389/fendo.2012.00031 PMC3355838 22645520

[15] Ely KA, Bischoff LA, Weiss VL. Wnt signaling in thyroid homeostasis and carcinogenesis. Genes (Basel). 2018;9(4):204. 10.3390/genes9040204 PMC5924546 29642644

[16] Xin S, Ye X. Knockdown of long non-coding RNA CCAT2 suppresses the progression of thyroid cancer by inhibiting the Wnt/β-catenin pathway. Int J Mol Med. 2020;46(6):2047–56. 10.3892/ijmm.2020.4761 PMC7595661 33125134

[17] Niehrs C. The complex world of WNT receptor signalling. Nat Rev Mol Cell Biol. 2012;13(12):767–79. 10.1038/nrm3470 23151663

[18] Chen W, Liu Z, Mai W, Xiao Y, You X, Qin L. FZD8 indicates a poor prognosis and promotes gastric cancer invasion and metastasis via B-catenin signaling pathway. Ann Clin Lab Sci. 2020;50(1):13–23. 32161008

[19] Ye Y, Li SL, Wang JJ. miR-100-5p downregulates mTOR to suppress the proliferation, migration, and invasion of prostate cancer cells. Front Oncol. 2020;10:578948. 10.3389/fonc.2020.578948 PMC7736635 33335853

[20] Takebayashi K, Nasu K, Okamoto M, Aoyagi Y, Hirakawa T, Narahara H. hsa-miR-100-5p, an overexpressed miRNA in human ovarian endometriotic stromal cells, promotes invasion through attenuation of SMARCD1 expression. Reprod Biol Endocrinol. 2020;18(1):31. 10.1186/s12958-020-00590-3 PMC7161200 32299427

[21] Bai D, Sun H, Wang X, Lou H, Zhang J, Wang X, et al. MiR-150 inhibits cell growth in vitro and in vivo by restraining the RAB11A/WNT/β-catenin pathway in thyroid cancer. Med Sci Monit. 2017;23:4885–94. 10.12659/MSM.906997 PMC5649516 29023429

[22] Li M, Qu L, Chen F, Zhu X. Propofol upregulates miR-320a and reduces HMGB1 by downregulating ANRIL to inhibit PTC cell malignant behaviors. Pathol Res Pract. 2020;216(4):152856. 10.1016/j.prp.2020.152856 32098696

[23] Li Q, Ye L, Zhang X, Wang M, Lin C, Huang S, et al. FZD8, a target of p53, promotes bone metastasis in prostate cancer by activating canonical Wnt/β-catenin signaling. Cancer Lett. 2017;402:166–76. 10.1016/j.canlet.2017.05.029 28602974

[24] Liu R, Chen Y, Shou T, Hu J, Qing C. miRNA-99b-5p targets FZD8 to inhibit non-small cell lung cancer proliferation, migration and invasion. Onco Targets Ther. 2019;12:2615–21. 10.2147/OTT.S199196 PMC6459141 31040702

[25] Sun S, Liu S, Duan SZ, Zhang L, Zhou H, Hu Y, et al. Targeting the c-Met/FZD8 signaling axis eliminates patient-derived cancer stem-like cells in head and neck squamous carcinomas. Cancer Res. 2014;74(24):7546–59. 10.1158/0008-5472.CAN-14-0826 25320014

[26] Chen F, Feng Z, Zhu J, Liu P, Yang C, Huang R, et al. Emerging roles of circRNA_NEK6 targeting miR-370-3p in the proliferation and invasion of thyroid cancer via Wnt signaling pathway. Cancer Biol Ther. 2018;19(12):1139–52. 10.1080/15384047.2018.1480888 PMC6301817 30207869

[27] Mao Y, Huo Y, Li J, Zhao Y, Wang Y, Sun L, et al. circRPS28 (hsa_circ_0049055) is a novel contributor for papillary thyroid carcinoma by regulating cell growth and motility via functioning as ceRNA for miR-345-5p to regulate frizzled family receptor 8 (FZD8). Endocr J. 2021;68(11):1267–81. 10.1507/endocrj.EJ21-0072 34108309

