# Supplementary material for: Overexpression of miR-100-5p inhibits papillary thyroid cancer progression via targeting FZD8
Source: Open Med (Wars). 2022 Jul 6;17(1):1172–82. doi: 10.1515/med-2022-0490 (PMC9263890; doi:10.1515/med-2022-0490)
Supplement: Supplementary Table [file med-2022-0490-sm.pdf]

Supplementary material

Table 1: Downstream molecules list of miR-100-5p

Target gene	Representative transcript	Gene name	Number of 3P-seq tags supporting UTR + 5	Link to sites in UTRs	Conserved sites				Poorly conserved sites				6mer sites	Representative miRNA
					total	8mer	7mer-m8	7mer-A1	total	8mer	7mer-m8	7mer-A1		
EPDR1	ENST00000199448.4	ependymin related 1	84	Sites in UTR	**1**	1	0	0	**1**	0	0	1	0	hsa-miR-100-5p
AP1AR	ENST00000274000.5	adaptor-related protein complex 1 associated regulatory protein	89	Sites in UTR	**1**	1	0	0	**0**	0	0	0	0	hsa-miR-99b-5p
ST6GALNAC4	ENST00000335791.5	ST6 (alpha-N-acetyl-neuraminyl-2,3-beta-galactosyl-1,3)-N-acetylgalactosaminide alpha-2,6-sialyltransferase 4	178	Sites in UTR	**1**	1	0	0	**0**	0	0	0	0	hsa-miR-99a-5p
TTC39A	ENST00000530004.1	tetratricopeptide repeat domain 39A	174	Sites in UTR	**1**	1	0	0	**0**	0	0	0	1	hsa-miR-99a-5p
HS3ST2	ENST00000261374.3	heparan sulfate (glucosamine) 3-O-sulfotransferase 2	5	Sites in UTR	**1**	1	0	0	**0**	0	0	0	0	hsa-miR-100-5p
BAZ2A	ENST00000379441.3	bromodomain adjacent to zinc finger domain, 2A	48	Sites in UTR	**2**	1	1	0	**0**	0	0	0	0	hsa-miR-99a-5p
KBTBD8	ENST00000295568.4	kelch repeat and BTB (POZ) domain containing 8	22	Sites in UTR	**1**	1	0	0	**0**	0	0	0	0	hsa-miR-100-5p
FKBP5	ENST00000536438.1	FK506 binding protein 5	32	Sites in UTR	**1**	1	0	0	**0**	0	0	0	0	hsa-miR-100-5p
HES7	ENST00000541682.2	hairy and enhancer of split 7 (Drosophila)	416	Sites in UTR	**1**	1	0	0	**0**	0	0	0	0	hsa-miR-99b-5p
HS3ST3B1	ENST00000360954.2	heparan sulfate (glucosamine) 3-O-sulfotransferase 3B1	1489	Sites in UTR	**1**	1	0	0	**0**	0	0	0	0	hsa-miR-99a-5p
FGFR3	ENST00000340107.4	fibroblast growth factor receptor 3	1085	Sites in UTR	**1**	1	0	0	**0**	0	0	0	0	hsa-miR-99a-5p
CTDSPL	ENST00000443503.2	CTD (carboxy-terminal domain, RNA polymerase II, polypeptide A) small phosphatase-like	372	Sites in UTR	**1**	1	0	0	**0**	0	0	0	0	hsa-miR-99b-5p
RAVER2	ENST00000371072.4	ribonucleoprotein, PTB-binding 2	218	Sites in UTR	**1**	0	1	0	**0**	0	0	0	0	hsa-miR-99a-5p
SMARCA5	ENST00000283131.3	SWI/SNF related, matrix associated, actin dependent regulator of chromatin, subfamily a, member 5	478	Sites in UTR	**1**	0	1	0	**0**	0	0	0	0	hsa-miR-100-5p
LRRC8B	ENST00000330947.2	leucine rich repeat containing 8 family, member B	326	Sites in UTR	**1**	1	0	0	**0**	0	0	0	0	hsa-miR-99a-5p
HOXA1	ENST00000355633.5	homeobox A1	157	Sites in UTR	**1**	0	1	0	**0**	0	0	0	0	hsa-miR-99a-5p
GMPS	ENST00000496455.2	guanine monphosphate synthase	867	Sites in UTR	**1**	1	0	0	**0**	0	0	0	0	hsa-miR-99a-5p
MTOR	ENST00000376838.1	mechanistic target of rapamycin (serine/threonine kinase)	653	Sites in UTR	**1**	0	1	0	**0**	0	0	0	0	hsa-miR-100-5p
NR6A1	ENST00000487099.2	nuclear receptor subfamily 6, group A, member 1	1082	Sites in UTR	**1**	1	0	0	**0**	0	0	0	0	hsa-miR-99b-5p
FZD8	ENST00000374694.1	frizzled family receptor 8	791	Sites in UTR	**1**	0	1	0	**0**	0	0	0	1	hsa-miR-100-5p
NTRK3	ENST00000394480.2	neurotrophic tyrosine kinase, receptor, type 3	7	Sites in UTR	**1**	1	0	0	**0**	0	0	0	0	hsa-miR-99a-5p
TMEM30A	ENST00000230461.6	transmembrane protein 30A	1221	Sites in UTR	**1**	0	0	1	**0**	0	0	0	0	hsa-miR-99a-5p
RASGRP3	ENST00000402538.3	RAS guanyl releasing protein 3 (calcium and DAG-regulated)	24	Sites in UTR	**1**	0	1	0	**0**	0	0	0	0	hsa-miR-100-5p
CLDN11	ENST00000064724.3	claudin 11	1116	Sites in UTR	**1**	0	1	0	**0**	0	0	0	0	hsa-miR-100-5p
TRIB2	ENST00000155926.4	tribbles pseudokinase 2	68	Sites in UTR	**1**	0	1	0	**0**	0	0	0	0	hsa-miR-100-5p
TRIB1	ENST00000311922.3	tribbles pseudokinase 1	313	Sites in UTR	**1**	0	0	1	**0**	0	0	0	0	hsa-miR-99b-5p
PPP3CA	ENST00000512215.1	protein phosphatase 3, catalytic subunit, alpha isozyme	259	Sites in UTR	**1**	0	1	0	**0**	0	0	0	0	hsa-miR-99a-5p
ZBTB7A	ENST00000322357.4	zinc finger and BTB domain containing 7A	37	Sites in UTR	**1**	0	0	1	**0**	0	0	0	1	hsa-miR-100-5p
GRHL1	ENST00000405379.2	grainyhead-like 1 (Drosophila)	147	Sites in UTR	**1**	0	0	1	**0**	0	0	0	0	hsa-miR-99a-5p
TMEM135	ENST00000340353.7	transmembrane protein 135	963	Sites in UTR	**1**	0	0	1	**0**	0	0	0	0	hsa-miR-100-5p
ST5	ENST00000526757.1	suppression of tumorigenicity 5	139	Sites in UTR	**1**	0	1	0	**0**	0	0	0	0	hsa-miR-100-5p
PPP1CB	ENST00000395366.2	protein phosphatase 1, catalytic subunit, beta isozyme	5795	Sites in UTR	**1**	0	0	1	**0**	0	0	0	0	hsa-miR-100-5p
NIPBL	ENST00000448238.2	Nipped-B homolog (Drosophila)	250	Sites in UTR	**1**	0	0	1	**0**	0	0	0	0	hsa-miR-99a-5p
MTMR3	ENST00000333027.3	myotubularin related protein 3	123	Sites in UTR	**1**	0	1	0	**0**	0	0	0	0	hsa-miR-99a-5p
PI15	ENST00000260113.2	peptidase inhibitor 15	12	Sites in UTR	**1**	0	0	1	**0**	0	0	0	0	hsa-miR-99a-5p
DESI2	ENST00000302550.11	desumoylating isopeptidase 2	147	Sites in UTR	**1**	0	0	1	**0**	0	0	0	0	hsa-miR-99a-5p
INSM1	ENST00000310227.1	insulinoma-associated 1	20	Sites in UTR	**1**	0	1	0	**0**	0	0	0	0	hsa-miR-99a-5p
CEP85	ENST00000252992.4	centrosomal protein 85kDa	471	Sites in UTR	**1**	0	0	1	**0**	0	0	0	0	hsa-miR-99a-5p
MBNL1	ENST00000357472.3	muscleblind-like splicing regulator 1	135	Sites in UTR	**1**	0	1	0	**0**	0	0	0	0	hsa-miR-99a-5p

(*Continued*)

Table 1: *Continued*

SMARCD1	ENST00000394963.4	SWI/SNF related, matrix associated, actin dependent regulator of chromatin, subfamily d, member 1	952	Sites in UTR	**1**	0	0	1	**0**	0	0	0	0	hsa-miR-99b-5p
NXF1	ENST00000531709.2	nuclear RNA export factor 1	1017	Sites in UTR	**1**	0	0	1	**0**	0	0	0	0	hsa-miR-100-5p
SLC44A1	ENST00000374720.3	solute carrier family 44 (choline transporter), member 1	407	Sites in UTR	**1**	0	1	0	**0**	0	0	0	0	hsa-miR-100-5p
ETV3	ENST00000368192.4	ets variant 3	37	Sites in UTR	**1**	0	1	0	**0**	0	0	0	0	hsa-miR-99a-5p
KDM6B	ENST00000254846.5	lysine (K)-specific demethylase 6B	8	Sites in UTR	**1**	0	1	0	**0**	0	0	0	0	hsa-miR-99b-5p
RRAGD	ENST00000369415.4	Ras-related GTP binding D	644	Sites in UTR	**1**	0	1	0	**1**	0	1	0	0	hsa-miR-100-5p
ZZEF1	ENST00000381638.2	zinc finger, ZZ-type with EF-hand domain 1	83	Sites in UTR	**1**	0	1	0	**0**	0	0	0	0	hsa-miR-100-5p
TRIM71	ENST00000383763.5	tripartite motif containing 71, E3 ubiquitin protein ligase	67	Sites in UTR	**1**	0	1	0	**0**	0	0	0	1	hsa-miR-99a-5p
CYP26B1	ENST00000001146.2	cytochrome P450, family 26, subfamily B, polypeptide 1	1315	Sites in UTR	**1**	0	1	0	**0**	0	0	0	0	hsa-miR-100-5p
CDYL2	ENST00000570137.2	chromodomain protein, Y-like 2	239	Sites in UTR	**1**	0	1	0	**0**	0	0	0	0	hsa-miR-99b-5p
IGF1R	ENST00000268035.6	insulin-like growth factor 1 receptor	561	Sites in UTR	**1**	1	0	0	**0**	0	0	0	0	hsa-miR-99b-5p
TAOK1	ENST00000261716.3	TAO kinase 1	164	Sites in UTR	**1**	1	0	0	**0**	0	0	0	0	hsa-miR-100-5p
THAP2	ENST00000308086.2	THAP domain containing, apoptosis associated protein 2	109	Sites in UTR	**1**	1	0	0	**0**	0	0	0	0	hsa-miR-100-5p
SATB1	ENST00000338745.6	SATB homeobox 1	312	Sites in UTR	**1**	1	0	0	**0**	0	0	0	0	hsa-miR-99b-5p
AGO2	ENST00000220592.5	argonaute RISC catalytic component 2	350	Sites in UTR	**1**	0	1	0	**0**	0	0	0	0	hsa-miR-100-5p
BMPR2	ENST00000374574.2	bone morphogenetic protein receptor, type II (serine/threonine kinase)	287	Sites in UTR	**1**	0	0	1	**0**	0	0	0	0	hsa-miR-100-5p
ZNRF2	ENST00000323037.4	zinc and ring finger 2	403	Sites in UTR	**1**	0	0	1	**0**	0	0	0	0	hsa-miR-100-5p
ICMT	ENST00000343813.5	isoprenylcysteine carboxyl methyltransferase	558	Sites in UTR	**1**	0	1	0	**0**	0	0	0	0	hsa-miR-99a-5p
RMND5A	ENST00000283632.4	required for meiotic nuclear division 5 homolog A (S. cerevisiae)	261	Sites in UTR	**1**	0	0	1	**0**	0	0	0	0	hsa-miR-99a-5p
FZD5	ENST00000295417.3	frizzled family receptor 5	122	Sites in UTR	**1**	0	1	0	**0**	0	0	0	0	hsa-miR-100-5p
